# Distributed Recovery of a Gaussian Source in Interference with Successive Lattice Processing

**DOI:** 10.3390/e21090845

**Published:** 2019-08-30

**Authors:** Christian Chapman, Matthew Kinsinger, Ameya Agaskar, Daniel W. Bliss

**Affiliations:** 1 School of Electrical, Computer and Energy Engineering, Arizona State University, Tempe, AZ 85281, USA; 2 MIT Lincoln Laboratory, Lexington, MT 02420, USA

**Keywords:** network information theory, distributed source coding, lattice codes

## Abstract

A scheme for recovery of a signal by distributed listeners in the presence of Gaussian interference is constructed by exhausting an “iterative power reduction” property. An upper bound for the scheme’s achieved mean-squared-error distortion is derived. The strategy exposes a parameter search problem, which, when solved, causes the scheme to outperform others of its kind. Performance of a blocklength-one scheme is simulated and is seen to improve over plain source coding without compression in the presence of many interferers, and experiences less outages over ensembles of channels.

## 1. Introduction

**Figure 1:**
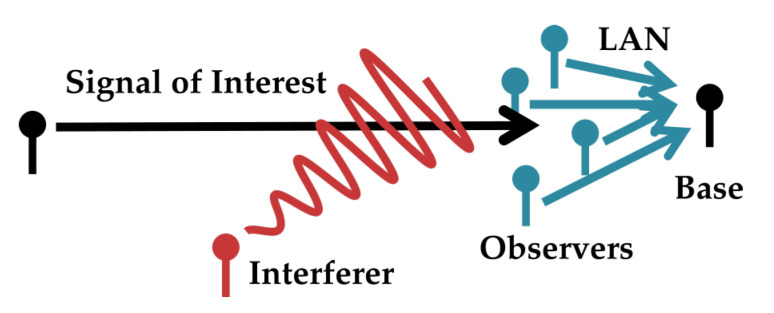
High-level view of the system. A base node seeks to recover a signal of interest to low mean-squared-error distortion in the presence of interference. The base receives information about the signal from several observer nodes. Observers encode their observations separately and forward a limited amount of information to the base over a LAN. The base uses this information to estimate the signal of interest. Note that in some scenarios the base could actually be a virtual node, representing all the messages any single observer has received over the LAN.

Consider a situation where multiple users have the common goal of recovering a broadcast in the presence of much stronger interfering signals. If the listening nodes are able to form a local area network (LAN) among one another, the nodes can mitigate interference by sharing information through the LAN and processing the messages they share. This scenario could arise, for instance, when a network of co-located nodes must aid a neighbor’s reception, when a group of cellular nodes have the common goal of receiving a code-domain-multiple-access transmission in a crowded environment, or when an adversary jams a broadcast node meant to serve multiple users. A high level model of this scenario is shown in Figure 1. When the amount of information transmission among listeners is limited (as it well may be if they must conserve power or bandwidth), they must take care to only forward novel information to their neighbors. This paper presents a lattice-algebra-based structured coding scheme for the observers in this scenario in an additive Gaussian noise channel with additive Gaussian interferers.

The strategy presented allows for strong recovery of the signal of interest in terms of mean-squared-error, even when the amount of information traded over the LAN is limited. It compares favorably to previous structured coding strategies of its kind, but still has a performance gap to random coding bounds in some regimes.

**Figure 2:**
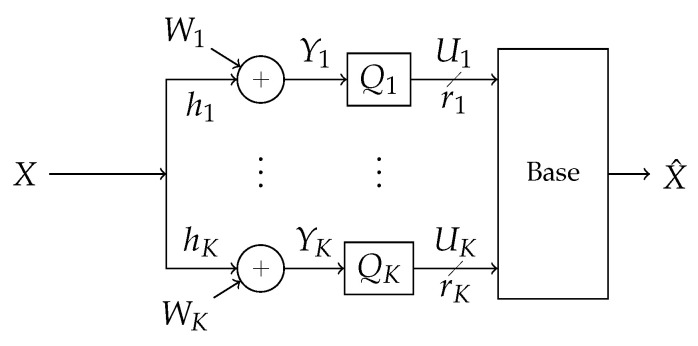
Block diagram of the system. A signal *X* is broadcast through an additive-white-Gaussian-noise channel in the presence of Gaussian interference, creating correlated additive noise $\left(W_{1},\ldots ,W_{K}\right)$. The signal is observed at *K* receivers labeled $Q_{1},\ldots ,Q_{K}$. The *k*th receiver observes $Y_{k}$ and processes its observation into a rate-$r_{k}$ message $U_{k}$. The messages are forwarded losslessly over a LAN to a base which processes the messages into an estimate of the signal, $\hat{X}$.

The scheme is stated in terms of a reduced version of the system where instead of communicating to one another, all listeners forward digital messages over a reliable LAN link to a base node which processes the messages into an estimate of the signal of interest. The LAN is modeled such that, for each channel use, each receiver forwards a fixed number of bits to the base. The LAN may be such that each receiver forwards to the base at the same rate, or the LAN may supports any receiver-to-base rates such that their sum-rate is below some maximum total throughput. This may arise if the LAN link is, e.g., an out-of-band frequency-domain-multiple-access channel where redistribution of receiver-to-base bitrates corresponds to redistribution of bandwidth resources. Both these situations are tested in Section 5. A more technical version of the model in Figure 1 is shown in Figure 2. The strategy is referenced in this paper as “Successive Integer-Forcing Many-help-one” (SIFM).

### 1.1. Relation to Other Literature

This is a strategy for the *Gaussian many-help-one source coding problem* [1], allowing for the case where the “receiver being helped” cannot provide side information to the decoder. The problem is also similar in structure to the CEO problem [2], but most studies on the CEO problem do not model correlated noise across observers. The rate-distortion region of this problem is achieved by random coding for some covariance structures [1], but is unknown in general. The rate-distortion region for the case of two helpers is known within bounds [3]. Unstructured coding schemes for the problem have long been known, although, given some results Wagner [4], Nazer and Gastpar [5] and Krithivasan and Pradhan [6], it seems likely that more structured coding strategies can outperform them in general.

The asymptotic version of the scheme is a direct application of general results and ideas from [7] to the many-help-one problem, although derivation differs, yielding a different characterization of its achieved rate region in terms of Algorithm 1. There is a lot of existing literature on lattice signal processing for this scenario, and similar techniques have been applied to closely related problems. The procedures which comprise SIFM have also been described notionally [8] and are contained within more general multiple-input-multiple-output (MIMO) communication studies [9] (Chapter 12). The authors are not aware of any direct presentation of this strategy for this problem until this work.

Integer Forcing Source Coding (IFSC) [8] is closely related to the present scheme. Further analytic properties of IFSC, simulations and robust configuration strategies for such codes are investigated in [10,11]. IFSC is extended to use the exhaustive power-reduction property in [12]. The studies just listed examine lattice techniques for a slightly different problem: design a coding strategy for the decoder to recover an estimate of all the observers’ signals, not just a single component of interest. Relaxing this constraint allows for a larger rate region for the many-help-one problem investigated here. Distributed-source-coding schemes such as IFSC and its related strategies provide weak solutions for the present problem as they intrinsically involve recovery of superfluous information. Likewise, the present scheme performs poorly for the IFSC distributed-source-coding problem as it produces a rank-deficient estimate of the sources.

SIFM is a strict generalization of a scheme by Krithvasan and Pradhan (KP) [13]. Although conceptually very similar, the generalizations of SIFM over KP necessitate a near ground-up re-description of the strategy. This parameterization of the KP scheme in terms of SIFM is shown in Appendix D. Unfortunately, SIFM replaces KP’s formulaic choice of certain parameters with a difficult continuous-domain nonlinear parameter search problem described in Section 5.2. This comes with the benefit of SIFM reliably outperforming KP, as shown in Section 5.

The lattice messages involved in such schemes are still non-trivially jointly distributed and could be further compressed to yield performance improvements Wagner [4], Yang and Xiong [14], Yang and Xiong [15] and Cheng et al. [16]. Most results on this correlation [4,14] focus on the case of two observers, and the compression described in [16] is inexhaustive. SIFM suffers from the same problem, and the amount of redundancy still present in observer messages has not yet been totally characterized. Analysis of the network scheme in the method presented here enables further study of these encoding redundancies. This will be shown in a forthcoming document.

### 1.2. Contributions

- A strategy for recovery of a Gaussian source using side information from many receivers in the presence of interference is presented in Section 3. This scheme outperforms others of its kind by exhausting a power-reduction property.
- An asymptotic version of the scheme is presented in Section 4, yielding Theorem 1.
- An algorithm for calculating the asymptotic scheme’s performance in Theorem 1 is presented as Algorithm 1.

### 1.3. Outline

The problem is set up analytically in Section 2. A table of symbols and notation is included in Table 1. Some algebraic properties of lattices are required to describe the strategy, and are reviewed briefly in Section 3. The scheme is described in terms of successful decoding events, which are constructed for arbitrary lattices in Definition 3. The definition yields a performance upper bound in Lemma 2. In limit with blocklength and certain choice of lattices, Lemma 2 yields an asymptotic performance in Corollary 1. This bound, along with several others, is plotted over various regimes in Section 5.

## 2. Problem Setup

A Gaussian source *X* is observed through a static channel by *K* receivers in the presence of additive Gaussian interference and noise, represented by a conglomerate term $W_{k}$. At each discrete time, receiver k∈[K] observes a real sample:

(1) $$Y_{k}:=h_{k}X+W_{k}$$

where $\left(h_{1},\ldots ,h_{K}\right)=\vec{h}\in R^{k}$, $\left(W_{1},\ldots ,W_{K}\right)=\vec{W}∼N\left(0,\Sigma_{W}\right)$, X∼N(0,1) and $X⊥\vec{W}.$ The above trial is repeated identically and independently *n* times to form time-expanded variables $X^{n}\in R^{n},\;{\vec{Y}}^{n},{\vec{W}}^{n}\in {\left(R^{n}\right)}^{K}$. A *base* node seeks to recover $X^{n}$ to low mean-squared-error.

### 2.1. Encoder

Receiver rates $r_{1},\ldots ,r_{K}>0$ are fixed. Receiver k∈[K] has an *encoder*${\mathrm{enc}}_{k}:R^{n}\to \left[2^{nr_{k}}\right]$, which forms an *encoding* $U_{k}={\mathrm{enc}}_{k}\left(Y_{k}^{n}\right)$. Each receiver forwards its encoding to the base perfectly.

### 2.2. Decoder

The base has a *decoder* $\mathrm{dec}:\prod_{k}\left[2^{nr_{k}}\right]\to R^{n}$ which processes the encodings $\left(U_{1},\ldots ,U_{K}\right)=\vec{U}$ into an estimate ${\hat{X}}^{n}:=\mathrm{dec}\left(\vec{U}\right)$.

### 2.3. Achievable Distortion

For rates ${\left(r_{k}\right)}_{k\in [K]}$ and noise covariance $\Sigma_{W}$, a distortion $d^{2}$ is *achievable* if there is a collection of encoders-and-decoder pairs $\left({\left({\mathrm{enc}}_{k}\right)}_{k\in [K]},\mathrm{dec}\right)$, the collection indexed by, say, *ℓ*, where $\inf_{\ell }\frac{1}{n}E\left[∥X^{n}-{\hat{X}}^{n}{∥}^{2}\right]\leq d^{2}$.

## 3. Successive Integer Forcing Many-Help-One Scheme

A block diagram of the scheme is shown in Figure 4. Broadly, the strategy operates as follows. *Design*: First, a blocklength *n* is chosen and a “coarse lattice” $L_{c}\subset R^{n}$ is chosen according to some design to be specified. One assumes the LAN is established and allows for reliable communication from receivers to the base node, each *k*th receiver at some rate $r_{k}$. Given these rates, a “fine lattice” $L_{k}⊃L_{c}$ is chosen for each *k*th receiver according to some design to be specified. At each *k*th receiver, a quantization dither $W_{d,k}$ is selected randomly uniformly over the base region of the receiver’s fine lattice, $L_{k}$.

Quantization in this scheme involves a dither term. The dither causes quantization noise to manifest as additive, independent of the input signal, and uniform over the base of $L_{k}$. These properties are demonstrated via the *crypto lemma* [9] (Theorem 4.1.1). Use of dither is described in detail in the Appendix proofs and must be included here for a complete description of the scheme’s operation but is inessential to an initial broad understanding of the scheme.

One assumes that the covariance between the transmitter’s signal and all the receivers’ observations is known at all the receivers and stable over *n* observations. As a function of rates $r_{1},\ldots ,r_{K}>0$ and the channel covariance matrix, some scale parameters $\alpha_{1},\ldots ,\alpha_{K}>0$ are designed.

*Operation:*

**Figure 3:**
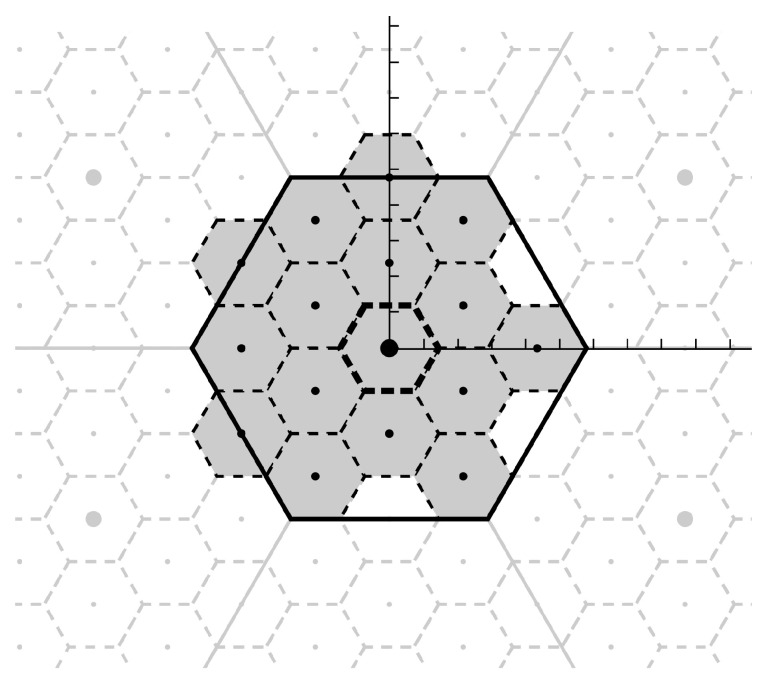
An example of nested lattices in $R^{2}$. Points from a coarse lattice $L_{c}$ are drawn as large dots. Points from a fine lattice $L_{k}⊃L_{c}$ besides those in $L_{c}$ are drawn as small dots. A base region $B_{c}$ for $L_{c}$ and its translates $B_{c}+L_{c}$ are outlined by solid lines, and a base region $B_{k}$ for $L_{k}$ and its translates $B_{k}+L_{k}$ are outlined by dashed lines. The base regions for points in $L_{k}\cap B_{c}$ are shaded. Notice that $L_{k}\cap B_{c}$ has $2^{4}$ points, so 4 bits are required to describe a general point in $L_{k}\cap B_{c}$, or 2 bits per sample.

**Figure 4:**
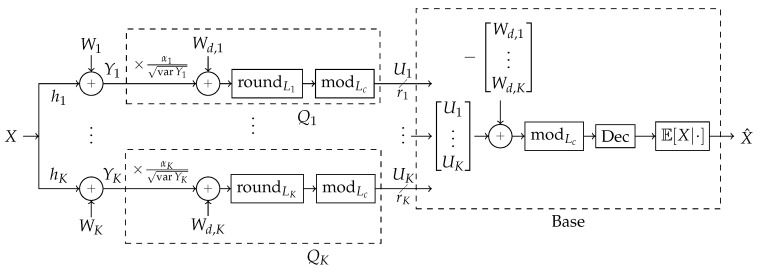
Block diagram of strategy, expanded from general block diagram in Figure 2. See the beginning of Section 3 for details.

1. Receiver *k*, labeled $Q_{k}$ in Figure 4, normalizes all its observed sequence of *n* samples by $\alpha_{k}/\sqrt{\mathrm{var}Y_{k}}$ so that expected-power-per-sample is 1.
2. Receiver *k* quantizes the result from the previous step by adding dither $W_{d,k}$, tamd hen rounding the result onto a nearby point on its fine lattice $L_{k}$.
3. Receiver *k* takes the modulo of the rounded result onto to $L_{c}$, producing a point in $L_{c}$’s “modulo-space”. $L_{k}$ must be designed with respect to $L_{c}$ such that the result of this step has entropy-rate less than $r_{k}$. Receiver *k* forwards this result to the base. See Figure 3 for an example design of $L_{c}$ and $L_{k}$ for blocklength n=2 and receiver rate $r_{k}=2$ bits per sample.
4. The decoder, labeled “base” in Figure 4, receives all the receiver messages, each message being some point in $L_{c}$-modulo-space, and removes the dither by subtracting the chosen dithers from each message, and taking the $L_{c}$-modulo of the result.
5. The decoder recombines them in a way that the result is no longer in modulo-space, but some linear combination of the quantizations.
6. The decoder recombines this recovered component from all the original modulo-space messages to produce new modulo-space points with the just-recovered component removed. The removal process is illustrated in Figure 6.
7. This removal allows for new recombinations to allow recovery of different components. This process is repeated until no more components can be recovered. All repetitions of Steps 5–7 are represented as the block labeled “Dec” in Figure 4.
8. All recovered components are used to estimate the source.

This is described in full precision below. To simplify exposition, from here on, assume $\mathrm{var}Y_{1}=\cdots =\mathrm{var}Y_{K}=1$ so that normalization is not necessary.

### 3.1. Background on Lattices

Before the scheme can be described, some preliminary definitions and statements are needed.

**Definition** **1.** *A* lattice *$L\subset R^{n}$ is an infinite set of discrete points closed under addition and subtraction.*

**Definition** **2.** *A region $s\subset R^{n}$ is a* base region *for a lattice L if the sets ${\left(l+s\right)}_{l\in L}$ are all disjoint, their union forms $R^{n},$ and if s has its moment at the origin.*

One can define modulo and rounding operations relative to a base region *s* for a lattice *L*:

(2) $$\begin{array}{cc} {\mathrm{round}}_{s} & :R^{n}\to L, \end{array}$$

(3) $$\begin{array}{cc} {\mathrm{round}}_{s}\left(x\right) & :=l\in L\;\mathrm{where}\;(x-l)\in s, \end{array}$$

(4) $$\begin{array}{cc} {\mathrm{mod}}_{s} & :R^{n}\to s, \end{array}$$

(5) $$\begin{array}{cc} {\mathrm{mod}}_{s}\left(x\right) & :=x-{\mathrm{round}}_{s}\left(x\right). \end{array}$$

**Figure 5:**
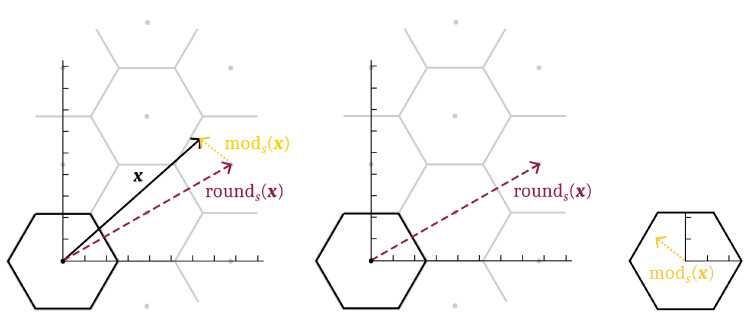
A graphical example of lattice modulo and rounding operations on a vector $x\in R^{2}$ for a particular lattice (drawn as dots) and hexagonal base region *s* (outlined in black, translates outlined by solid gray lines). Notice $x={\mathrm{round}}_{s}\left(x\right)+{\mathrm{mod}}_{s}\left(x\right)$, that the image of ${\mathrm{round}}_{s}\left(\cdot \right)$ is the lattice, and that the image of ${\mathrm{mod}}_{s}\left(\cdot \right)$ is *s*.

Note that ${\mathrm{round}}_{s}$ is well defined since *s* being a *base region* implies there is one and only one lattice point *l* that satisfies its prescription. These operations have some useful properties. A graphical example of the lattice round and modulo operations for a lattice in $R^{2}$ and a hexagonal base region *s* is shown in Figure 5.

**Property** **1.** *(mod is the identity within s) x∈s has ${\mathrm{mod}}_{s}\left(x\right)=x$.*

**Proof.**  ${\mathrm{mod}}_{s}\left(x\right)=x-{\mathrm{round}}_{s}\left(x\right)=x.$ □

**Property** **2.** *$x\in R^{n},l\in L$ have ${\mathrm{round}}_{s}\left(x+l\right)={\mathrm{round}}_{s}\left(x\right)+l.$*

**Proof.**  $\left(x+l\right)-\left({\mathrm{round}}_{s}\left(x\right)+l\right)=\left(x-{\mathrm{round}}_{s}\left(x\right)\right)\in s.$ Thus, by definition, ${\mathrm{round}}_{s}\left(x+l\right)={\mathrm{round}}_{s}\left(x\right)+l.$ □

**Property** **3.** *$x\in R^{n},l\in L$ have ${\mathrm{mod}}_{s}\left(x+l\right)={\mathrm{mod}}_{s}\left(x\right).$*

**Proof.**  ${\mathrm{mod}}_{s}\left(x+l\right)=\left(x+l\right)-{\mathrm{round}}_{s}\left(x+l\right)=\left(x+l\right)-{\mathrm{round}}_{s}\left(x\right)-l=x-{\mathrm{round}}_{s}\left(x\right)={\mathrm{mod}}_{s}\left(x\right).$ The second equality is by Property 2. □

**Property** **4.** (Lattice modulo is distributive) *$x,y\in R^{n}$ have:*

(6) $$\begin{array}{cc} {\mathrm{mod}}_{s}\left({\mathrm{mod}}_{s}\left(x\right)+y\right) & ={\mathrm{mod}}_{s}\left(x+y\right). \end{array}$$

**Proof.**  ${\mathrm{mod}}_{s}\left({\mathrm{mod}}_{s}\left(x\right)+y\right)={\mathrm{mod}}_{s}\left(x+y-{\mathrm{round}}_{s}\left(x\right)\right)={\mathrm{mod}}_{s}\left(x+y\right).$ The last equality is by Property 3. □

**Figure 6:**
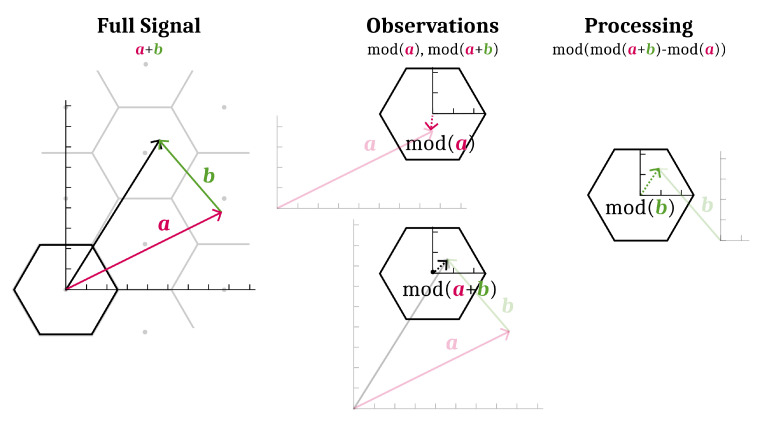
Removal of a vector component a from modulo mod(a+b). Drawn in the same context as Figure 5. The full signal, a+b without any modulo operations, is plotted on the left. Say that available for processing are the vectors mod(a) and mod(a+b), illustrated in the middle. A particular processing of mod(a) and mod(a+b) produces mod(b), shown on the right. Non-modulo components are drawn lightly under each modulo.

An example of how Property 4 can be used is shown in Figure 6. The efficacy of the lattice strategy presented in this paper is in exhaustive use of this technique.

### 3.2. Lattice Scheme Description

Fix the following parameters:

- Blocklength *n*
- Receiver scales $\vec{\alpha }=\left(\alpha_{1},\ldots ,\alpha_{K}\right)\in R^{K}$
- “Fine” lattices $L_{k}\subset R^{n}$ each with a base region $B_{k}\subset R^{n}$, k∈[K]
- A “coarse” lattice $L_{c}\subset R^{n}$ with a base region $B_{c}$, where $L_{k}⊃L_{c}$ and $\frac{1}{n}\log\left|B_{c}\cap L_{k}\right|\leq r_{k}$ for each k∈[K].
- Functions $\phi_{k}:\left(B_{c}\cap L_{k}\right)\to \left[2^{nr_{k}}\right]$ which enumerate their domain’s points
- Dither variables ${\vec{W}}_{d}=\left(W_{d,1},\ldots ,W_{d,K}\right)$, with $W_{d,k}∼\mathrm{unif}B_{k}$ independent over *k*

For brevity we neglect to denote application of the enumeration $\phi_{k}$ and its inverse when it is clear from context where it should be applied.

#### 3.2.1. Encoders

Each encoder first scales its observation then quantizes the result by rounding with dither onto its fine lattice $L_{k}$. This discretizes the source’s observation onto a countable collection of points, but it may still be too high-rate to forward to the base directly. The encoder wraps the discretization onto the coarse lattice’s base region $B_{c}$ by applying ${\mathrm{mod}}_{B_{c}}$. The domain reduction from all of $L_{k}$ to only points within $B_{c}\cap L_{k}$ reduces the discretization’s entropy enough to forward it to the decoder.

Construct the encoder for receiver *k* as ${\mathrm{enc}}_{k}:R^{n}\to \left[2^{nr_{k}}\right]$:

(7) $$\begin{array}{cc} {\mathrm{enc}}_{k}\left(y_{k}^{n}\right) & :=\phi_{k}\left({\mathrm{mod}}_{L_{c}}\left({\mathrm{round}}_{L_{k}}\left(\alpha_{k}y_{k}^{n}+W_{d,k}\right)\right)\right) \end{array}$$

#### 3.2.2. Decoders

The decoder produces estimates of particular integer linear combinations $a_{1},\ldots ,a_{K}$ of the source observations by processing the encodings in stages. In stage *k*, the decoder recovers combination $a_{k}$. In all future stages, the $a_{k}$ component is used to aid recovery. This is described in more detail below. To construct each stage, it is necessary to describe the covariance between receiver quantizations. By [9] (Theorem 4.1.1),

(8) $${\mathrm{round}}_{L_{k}}\left(\alpha_{k}Y_{k}^{n}+W_{d,k}\right)-W_{d,k}=\alpha_{k}Y_{k}^{n}-{\tilde{W}}_{d,k}$$

and ${\tilde{W}}_{d,k}∼\mathrm{unif}B_{k}$ is independent of $Y_{k}^{n}$. Denote ${\tilde{W}}_{d}=\left({\tilde{W}}_{d,1},\ldots ,{\tilde{W}}_{d,K}\right)$. In addition, denote the scaled receiver observations as $\vec{\alpha Y}=\left(\alpha_{1}Y_{1},\ldots ,\alpha_{K}Y_{K}\right),$ and similarly for time-expanded ${\vec{Y}}^{n}.$ Then, on average over time, the receivers’ dithered rounding to their fine lattices effectively adds noise of the following covariance to the observations:

(9) $$\begin{array}{c} \Sigma_{Q}:=\mathrm{diag}\left({\left(E\left[\frac{1}{n}{∥{\tilde{W}}_{d,k}∥}^{2}\right]\right)}_{k\in [K]}\right). \end{array}$$

A covariance matrix C between the sources after dithered rounding to the fine lattices can be written as the time-averaged covariance of vectors in Equation (8):

(10) $$\begin{array}{cc} C & :=E\left[\frac{1}{n}{\left({\vec{\alpha Y}}^{n}-{\tilde{W}}_{d}\right)}^{†}\left({\vec{\alpha Y}}^{n}-{\tilde{W}}_{d}\right)\right]. \end{array}$$

(11) $$\begin{array}{cc} \; & =\left(\mathrm{diag}\vec{\alpha }\right)\Sigma {\left(\mathrm{diag}\vec{\alpha }\right)}^{†}+\Sigma_{Q}. \end{array}$$

Now, some events over outcomes of $\left({\vec{\alpha Y}}^{n}-{\tilde{W}}_{d}\right)$ are constructed.

**Definition** **3.** *For some $K^{\prime }\in \left[K\right]$, fix an integer matrix $A\in Z^{K\times K^{\prime }}$, call its columns $a_{1},\ldots ,a_{K^{\prime }}\in Z^{K},$ and take $A_{k}$ to be the first k columns of A.*

*In terms of A, define matrices for each $k=1,\ldots ,K^{\prime }:$*

(12) $$\begin{array}{cc} S_{k}\left(v\right) & :=\underset{u\in R^{k}}{\arg\min}\mathrm{var}\left({\left(v-A_{k}u\right)}^{†}\left({\vec{\alpha Y}}^{n}-{\tilde{W}}_{d}\right)\right) \end{array}$$

(13) $$\begin{array}{cc} R_{k}\left(v\right) & :=[I_{K\times K}-A_{k}S_{k}]v. \end{array}$$

*The argmin above derived in closed form in Appendix C.*

*In addition, in terms of A, define events $M\left(A_{1}\right),\ldots ,M\left(A_{K^{\prime }}\right)\subset {\left(R^{n}\right)}^{K}$:*

(14) $$\begin{array}{cc} M(A_{1}) & :=\{z\in R^{K\times n}:a_{1}^{†}z\in B_{c}\}, \end{array}$$

(15) $$\begin{array}{cc} M(A_{k}) & :=M\left(A_{k-1}\right)\cap \left\{z\in R^{K\times n}:{\left(R_{k-1}a_{k}\right)}^{†}z\in B_{c}\right\}. \end{array}$$

The events designate when a particular processing of the encodings successfully produces an estimate of the observations *without modulo*:

**Lemma** **1.** *Fix $A\in Z^{K\times K^{\prime }}$ as in Definition 3. Take $U_{k}={\mathrm{enc}}_{k}\left(Y_{k}^{n}\right)$ for k∈[K]. There is a function f where $f\left(\vec{U}\right)=A^{†}\left({\vec{\alpha Y}}^{n}-{\tilde{W}}_{d}\right)$ whenever $\left({\vec{\alpha Y}}^{n}-{\tilde{W}}_{d}\right)\in M\left(A\right)$.*

*The functions $f_{1},\ldots ,f_{K^{\prime }}$ for each output of f, $f_{\left[k\right]}$ denoting the first k outputs, are given as follows, all *mod* taken with respect to coarse base region $B_{c}$.*

(16) $$\begin{array}{cc} f_{1}\left(\vec{U}\right) & :=\mathrm{mod}\left(a_{1}^{†}\left(\vec{U}-{\vec{W}}_{d}\right)\right), \end{array}$$

(17) fmU→:=mod∑abam†(U→−W→d)−…mod[Sm−1am]†f[m−1](U→)+…[Sm−1am]†f[m−1](U→).

The proof is delayed to Appendix A.

This aspect of general reuse of all previously recovered components for the recovery of a new one is the source of benefit of the present scheme over [13]. Comments in [9] among other places describe such a strategy.

A decoder can be realized from each event from Definition 3 by using a linear estimator on the output of *f* gotten from that event in Lemma 1. The likelihood of each event is quite sensitive to channel covariance Σ, receiver scalings $\vec{\alpha }$ and integer vector $a_{\ell }$. As layers of component recovery are added, the events become increasingly unlikely. Thus, only a few decoders perform reliably.

#### 3.2.3. Decoder Performance

We now bound the worst-case performance of such decoders.

**Definition** **4.** *In the context of Lemma 1, take $e_{X|A}$ to be the coefficients of the best linear unbiased estimator for X given $A^{†}\left(\vec{\alpha Y}-{\vec{W}}_{Q}\right),\;{\vec{W}}_{Q}∼N\left(0,\Sigma_{Q}\right)$. Then, for $\Delta >\mathrm{var}\left(X|A^{†}\left(\vec{\alpha Y}-{\vec{W}}_{Q}\right)\right)$, define a decoder:*

(18) $$\begin{array}{cc} & \mathrm{dec}\left(\vec{U};A\right):=e_{X|A}^{†}f\left(\vec{U}\right), \end{array}$$

*or 0 if the observed average power of Equation (18) is greater than* Δ.

The mean-squared-error distortion each such decoder achieves can be upper bounded in terms of the probability of the event M(A):

**Lemma** **2.** *Take a decoder from Definition 4 and define*

(19) $$d^{2}:=1-\mathrm{var}\left(X|e_{X|A}^{†}\left(\vec{\alpha Y}+{\vec{W}}_{Q}\right)\right).$$

*Take E to be the event where the decoding is zero. Then, no worse than the following mean-squared-error is achieved in estimating $X^{n}$:*

(20) $$\begin{array}{c} d^{2}+{\left(\Delta +1\right)}^{2}\cdot \sqrt{3P(M^{C})}+\sqrt{3P(E)} \end{array}$$

**Proof.**  The goal is to approximate the integral:

(21) $$\begin{array}{cc} \int \frac{1}{n}{∥\mathrm{dec}\left(\vec{U};A\right)-X^{n}∥}^{2}dP & =\int_{M}\frac{1}{n}{∥\mathrm{dec}\left(\vec{U};A\right)-X^{n}∥}^{2}dP+\ldots \\ \; & \;\int_{M^{C}}\frac{1}{n}{∥\mathrm{dec}\left(\vec{U};A\right)-X^{n}∥}^{2}dP. \end{array}$$

Bound the first summand of Equation (21):

(22) $$\begin{array}{cc} \int_{M}\frac{1}{n}{∥\mathrm{dec}\left(\vec{U};A\right)-X^{n}∥}^{2}dP & =\frac{1}{n}\int_{M}1_{E^{C}}∥E\left[X^{n}|A\left(\vec{\alpha Y}-{\vec{W}}_{Q}\right)\right]-X^{n}{∥}^{2}+1_{E}{∥X^{n}∥}^{2}dP \end{array}$$

(23) $$\begin{array}{cc} \; & <\frac{1}{n}\int ∥E\left[X^{n}|A\left(\vec{\alpha Y}-{\vec{W}}_{Q}\right)\right]-X^{n}{∥}^{2}dP+\frac{1}{n}\int_{E}{∥X^{n}∥}^{2}dP \end{array}$$

(24) $$\begin{array}{cc} \; & =d^{2}+\frac{1}{n}\int_{E}{∥X^{n}∥}^{2}dP. \end{array}$$

where the first equality follows by Lemma 1 and choice of decoder. Bound the second summand of Equation (21):

(25) $$\begin{array}{cc} \int_{M^{C}}\frac{1}{n}{∥\mathrm{dec}\left(\vec{U};A\right)-X^{n}∥}^{2}dP & \leq \frac{1}{n}\int_{M^{C}}{∥\left(1+\Delta \right)X^{n}∥}^{2}dP \end{array}$$

(26) $$\begin{array}{cc} \; & ={\left(1+\Delta \right)}^{2}\frac{1}{n}\int_{M^{C}}{∥X^{n}∥}^{2}dP. \end{array}$$

Applying Hölder’s inequality as follows to the two bounds yields the result:

(27) $$\begin{array}{cc} \int_{S}∥X^{n}∥dP & \leq {\left[\int 1_{S}^{2}dP\right]}^{1/2}\cdot {\left[{\int ∥X∥}^{4}dP\right]}^{1/2} \end{array}$$

(28) $$\begin{array}{cc} \; & =\sqrt{3P(S)}. \end{array}$$

□

The bound on distortion suffices for the asymptotic analysis in Section 4, but is quite coarse in low dimension.

## 4. Asymptotic Scheme

Analysis of the scheme in limit with blocklength over particular choice of $L_{c},{\left(L_{k}\right)}_{k\in [K]}$ yields a nice characterization of its performance. Theorem 1 demonstrates that, in limit with blocklength and certain lattice design, there is essentially one decoder which performs at least as well as any others. The subspace of receiver observations it reliably recovers is characterized. First, a matrix definition is needed to state this result.

**Definition** **5.** *For ε>0 define covariance matrices $\Sigma_{Q\infty },C_{\infty }\in R^{K\times K}$*

(29) $$\begin{array}{cc} \Sigma_{Q\infty } & :=\mathrm{diag}\left(2^{-2r_{1}+\varepsilon },\ldots ,2^{-2r_{K}+\varepsilon }\right). \end{array}$$

(30) $$\begin{array}{cc} C_{\infty } & :={\left(\mathrm{diag}\vec{\alpha }\right)}^{†}\Sigma \left(\mathrm{diag}\vec{\alpha }\right)+\Sigma_{Q\infty }. \end{array}$$

$C_{\infty }$ represents the covariance between quantized observations achieved in limit with blocklength when the lattices ${\left(L_{k}\right)}_{k\in [K]}$ are chosen well.

**Theorem** **1.** *Take ε>0 small and $C_{\infty }$ from Equation (29). Define *S* to be the smallest subspace in $R^{K}$ with the property that all integer vectors $v\in Z^{K}$ have either $\min_{s\in S}{\left(v-s\right)}^{†}C_{\infty }\left(v-s\right)\geq 1$ or v∈S. Fix $P_{\infty }$ as the projection onto S. Then, for large enough blocklength n and certain encoders, some processing f of the encodings has with high probability*

(31) $$f\left(\vec{U}\right)=P_{\infty }\left({\vec{\alpha Y}}^{n}+{\vec{W}}_{Q}^{\prime }\right),$$

*where ${\vec{W}}_{Q}^{\prime }=\left(W_{Q,1}^{\prime },\ldots W_{Q,K}^{\prime }\right)$ has independent components and $E(\frac{1}{n}∥W_{Q,k}^{\prime }{∥}^{2})<2^{-2r_{k}+2\varepsilon }.$*

**Corollary** **1.** *A decoder provided side information as in Theorem 1 can achieve the following MSE distortion in estimating X:*

(32) $$d_{L}^{2}:=1-{\vec{\alpha }}^{†}\mathrm{pinv}\left(P_{\infty }C_{\infty }P_{\infty }\right)\vec{\alpha }.$$

**Proof.**  Apply a linear estimation for the source on *f*’s output. □

The proof is given in Appendix B. The theorem is demonstrated by observing that, if lattices are chosen well, then events from Definition 3 approach probability zero or one, and that $P_{\infty }$’s image coincides with the span of the high-probability-events’ vectors. Computation of the projection $P_{\infty }$ can be done via repeated reduction of $C_{\infty }^{1/2}:$

**Algorithm 1** Compute projection $P_{\infty }$ and processing stages A from $C_{\infty }$.

$A\leftarrow \left[\;\right],\;a\leftarrow \mathrm{SLVC}\left(C_{\infty }^{1/2}\right),\;R\leftarrow I_{K\times K},$

**while** $0<{\left(Ra\right)}^{†}C_{\infty }Ra<1$ **do**
    A←[A,a]    $R\leftarrow I_{K\times K}-A\mathrm{pinv}\left(A^{†}C_{\infty }A\right)A^{†}C_{\infty }$    $a\leftarrow \mathrm{SLVC}(C_{\infty }^{1/2}R)$
**end while**

$P_{\infty }\leftarrow A{\left(A^{†}A\right)}^{-1}A^{†}$

**return** $P_{\infty },\;A$



In Algorithm 1, the subroutine SLVC(B), “Shortest Lattice Vector Coordinates” returns the nonzero integer vector a that minimizes the norm of Ba while Ba≠0. SLVC(·) can be implemented using a lattice enumeration algorithm like one in [17] together with the Lenstra-Lenstra-Lovász (LLL) algorithm to convert a set of spanning lattice vectors into a basis [18]. Algorithm 1 indeed returns $P_{\infty }$ since it is lifted from $P_{\infty }$’s construction in the proof of Theorem 1 given in Appendix B.

### Complexity of Scheme

The complexity of the scheme in operation is identical to that of codes with similar structure. Examples include IFSC and KP, as discussed in the Introduction. In particular, if g(n) grows with the amount of operations used to evaluate ${\mathrm{round}}_{L}$ (*L* being the involved lattice for which ${\mathrm{round}}_{L}$ is hardest to compute), then it follows from the scheme description that the time complexity of each encoder is O(n+g(n)) and the time complexity of the base’s decoder is $O(nK+K^{2}\cdot g\left(n\right)))$. The practicality of such a scheme is then primarily dependent on the existence of lattices which both satisfy the “goodness” properties described here and have rounding, modulo operations of tractable complexity. Several propositions for such lattice structures exist, for instance *LDPC lattices* (O(n)) [19] and *polar lattices* (O(nlogn)) [20].

Configuration of the scheme is also a difficult computational problem. Determination of the optimal configuration is a non-smooth continuous-domain search for the objective given in Corollary 1 over choice of encoder scalings $\vec{\alpha }\in R^{+}$. Each objective evaluation involves computing Algorithm 1. Due to this algorithm involving several shortest-lattice-vector-problems in dimension up to *K*, one implementation of this algorithm has time complexity $O(K2^{K})$. This complexity reduces considerably if the strict shortest-lattice-vector problem is relaxed to allow approximate solutions such as those provided by the LLL algorithm. No methods for global optimization of the objective are known, but computationally tractable approximations can provide strong performance as demonstrated in Section 5.

## 5. Performance

Here, the performance of the scheme is compared to existing results. All numerical results deal with complex circularly-symmetric Gaussian channels. Although the SIFM derivations shown are in terms of real channels for easier exposition, they apply just as well to the complex case by considering each complex observation as two appropriately correlated real observations. Each data point was computed on average over 200 randomly generated channel covariances selected with the prescribed statistics. An outer bound, representing the performance if the base had access to the receivers’ observations in full precision, is also shown.

### 5.1. Quantize and Forward

One strategy much simpler than SIFM is for each observer forward its own quantized representation of its observations to the base. A “saturating uniform quantizer” was simulated, where each real observation was clipped to some interval, and then rounded onto a quantization step using the prescribed data rate. This curve is labeled “1D Quantize and Forward”.

Performance when observers used rate-distortion quantizers for Gaussian sources was also computed and is labeled “HD Quantize and Forward”. The performance for the rate-distortion quantizer strategy when observer bitrates were allowed to vary within a sum-rate was also plotted as “HD Quantize and Forward (Variable-Bitrate)”.

**Figure 7:**
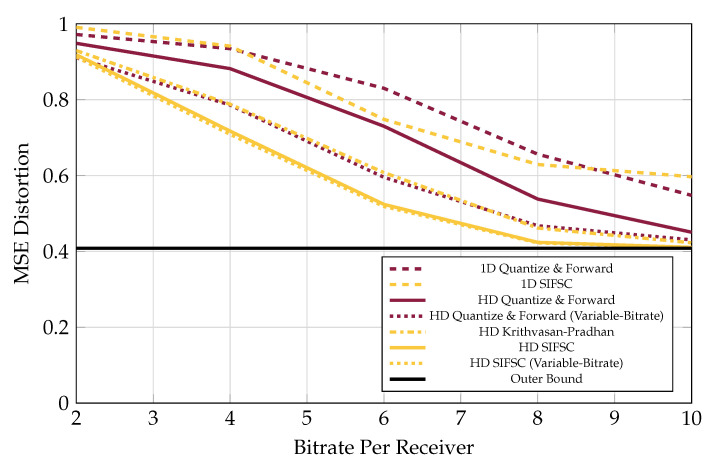
MSE performance versus receiver bitrate for five receivers the presence of three 20 dB interferers, all observing a 0 dB source. SIFM consistently significantly outperforms the rest of the schemes, and does not appear to benefit much from redistribution of observer bitrates.

**Figure 8:**
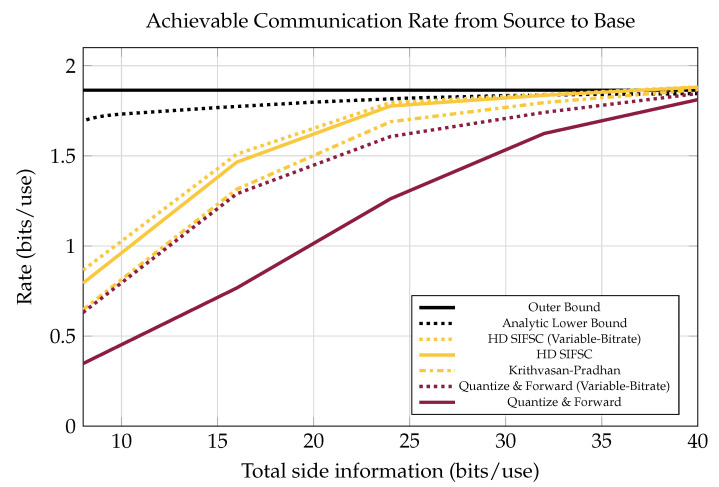
Achievable end-to-end communication rate between source and base for various sources, including an achievable rate using Berger-Tung-like lossy distributed source coding. Four receivers each observing a source at 0 dB in the presence of one 20 dB interferer. Notice that at low bitrates there is a large gap between this bound and the achieved SIFM rate. This could either be due to the fact that SIFM messages are not independent of each other and could be further jointly compressed, or because the SIFM messages are sub-optimally configured.

**Figure 9:**
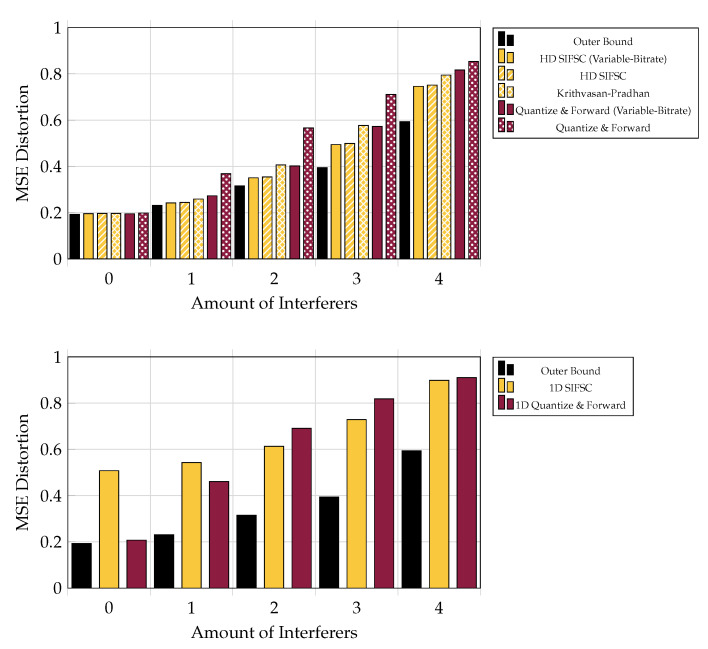
MSE performance versus amount of 20 dB interferers, when each of five observer encodes at a rate of 6 bits per observation (or 30 bits total shared among observers for Variable-Rate strategies). SIFM consistently outperforms the rest of the strategies and was not observed to improve much by reconfiguring observer bitrates. One-shot SIFM did not consistently outperform much simpler one-shot quantizers.

**Figure 10:**
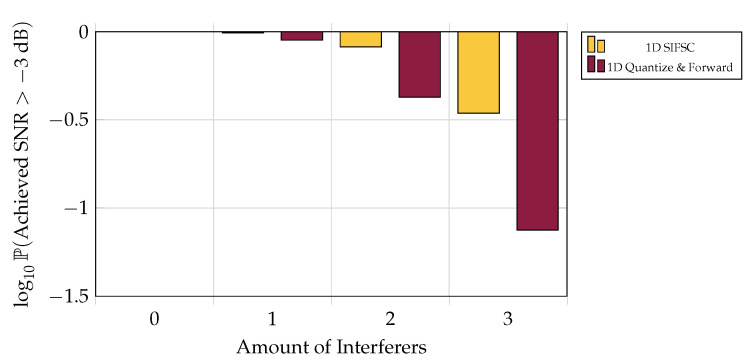
Likelihood of scheme providing recovery of *X* better than −3 dB SNR versus amount of 20 dB interferers, when each of five observer encodes at a rate of 6 bits per observation. For this threshold and ensemble of channels, one-shot SIFM performs roughly as reliably as one-shot Quantize and Forward with one less 20 dB interferer present.

These techniques are strong when the LAN rate is severely limited or the interference is low, as shown in Figures 7 and 9. The function of joint compression in these regimes is more subtle since there are less superfluous correlated components among receiver observations to eliminate. Quantize and Forward schemes are weak relative to the others in the presence of many strong interferers, as shown in Figure 7, Figure 8, Figure 9 and Figure 10.

### 5.2. Asymptotic Scheme

The distortion SIFM achieves is dependent on choice of observer scales $\vec{\alpha }$ and is highly nonconvex in terms of them due to the discontinuity of Algorithm 1’s outputs. For this reason, some form of search for good scale parameters is required for each fixed channel covariance and observer rate vector $\vec{r}$. For performance evaluation, the search problem was solved approximately.

Recall that the asymptotic scheme described here includes one by Krithivasan and Pradhan (KP, see Introduction) as a special case. Details of the parameterization of the KP scheme in terms of SIFM are shown in Appendix D. It was observed empirically that scaling all receiver’s observation by the same constant can yield strong performance. The strongest between uniform and KP scaling was chosen as an initial guess and was improved by taking random steps.

### 5.3. One-Shot Scheme

If the involved lattices are all one-dimensional (i.e., nested intervals), then the likelihood of each event in Definition 3 is straightforward to approximate via Monte-Carlo simulation. This enables a slight modification of Algorithm 1 to be used for identifying a strong decoding strategy for given receiver scalings. Although the upper bound in Lemma 2 applies to the one-shot strategy, it is often too weak in low dimension to be informative of a scheme’s true distortion. Distortion was estimated by simulation instead. In plots, this scheme is labeled “1D SIFM”.

Unfortunately, the best identified configurations for the one-shot scheme did not consistently provide significant improvement over uniform quantization as the high dimensional scheme had. This is thought to be because, even in extreme observations, the uniform quantizer saturates and still provides a reasonable representation of the source. In contrast, the same observation in SIFM creates a lattice wrapping distortion, which significantly affects the final decoding result. Reducing the likelihood of such errors in low dimension requires conservative choice of scales, which further degrades performance. However, such problems diminish as higher dimensional lattices are used.

Performance for the asymptotic SIFM strategy when observer bitrates were allowed to vary within a sum-rate was also plotted as “HD SIFM (Variable-Bitrate)”.

### 5.4. Versus Increasing Receiver Rates

The performance of the various schemes considered is shown in Figure 7 for five receivers, each messaging to the base at rate varying from 2 bits-per-complex-sample u to 16 bits-per-complex-sample, in the presence of three interferers each appearing at each observer at an average of 20 dB above a 0 dB signal of interest.

Coincidentally, the performance of the KP scheme with equal bitrates was observed in all plots to closely match the performance of the variable-bitrate Quantize and Forward scheme.

As shown in Figure 8, there is still a significant gap at low bitrates between the performance of SIFM and the bound given in [21]. This bound is based on non-structured joint-compression of quantizations (like Berger-Tung source coding [22] but binned for recovery of a source rather than all the observers’ quantizations). The gap in performance could be due to a combination of factors. First, the scheme from [21] is designed so that receiver messages are independent of one another, while in SIFM some inter-message dependences are still present after lattice processing. This means SIFM messages could be jointly compressed to improve performance. Another contributing factor could be poor solution of the search problem mentioned in Section 5.2.

### 5.5. Versus Adding Interferers

Performance for the various schemes is shown in Figure 9 for a system of five receivers, each messaging to the base at a rate of 6 bits-per-complex-sample, in the presence of 0–5 independent interferers, each appearing at each observer an average of 20 dB above a 0 dB signal of interest.

### 5.6. One-Shot Outage Probability

One may be interested in the likelihood of being able to recover a signal to above some acceptable noise threshold over an ensemble of channels. Although in low-interference regimes the one-shot SIFM scheme is reliably outperformed by the much simpler one-shot Quantize and Forward scheme (Figure 9), one-shot SIFM is much less likely to perform exceptionally poorly when averaged over the current channel model (see Figure 10).

## 6. Conclusions

Successive Integer-Forcing Many-Help-One (SIFM) is a lattice-algebra-based strategy for distributed coding of a Gaussian source in correlated noise. For good choice of parameters, SIFM consistently outperforms many of the strategies it generalizes. Finding good parameters for SIFM is a difficult non-convex search problem but reasonably strong solutions can be found through well-initialized random search.

A one-shot implementation of the scheme usually outperforms plain uncompressed quantization when multiple interferers are present, but often performs worse when there are few. This is probably due to SIFM’s heavy dependence on the absence of tail events that are somewhat common in low blocklength versions of the scheme. Despite this, the one-shot scheme is more typically above low-SNR thresholds in certain ensembles of channels. It is expected that performance would improve greatly if higher dimensional nested lattices were used.

Many results for related lattice schemes remain to be ported to the present case. Configurations for IFSC encoders robust to incomplete channel state knowledge are designed in Reference [10] (Chapter 4). Robustness of such schemes of IFSC to Doppler and delay spread in a certain system model are investigated in Griffin [11].

There is still some gap between the best achievable rate and SIFM, as seen in Figure 8. This is at least partially due to redundancies in SIFM messages. Some redundancies still exist between SIFM messages Wagner [4] Yang and Xiong [14]. This indicates that further compression of the messages is possible, and a study on these redundancies is forthcoming.

## Figures and Tables

**Table 1:** Symbols and notation.

[n]	Whole numbers from 1 to *n*
$A,a,\vec{a},\vec{A}$	Matrix, column vector, vector, random vector
$A^{†},a^{†}$	Hermitian conjugate
$\mathrm{diag}\vec{a}$	Square diagonal matrix with diagonals $\vec{a}$
pinv(·)	Moore-Penrose pseudoinverse
P(·)	Current context’s unconditioned probability measure
N(0,Σ)	Normal distribution with zero mean, covariance Σ
X∼f	*X* is a random variable distributed like *f*
var(X)	Variance (or covariance matrix) of *X*
$X^{n},f\left(x^{n}\right)$	Vector of *n* independent trials of a random variable distributed like *X*, a function whose input is intended to be such a variable
var(a|b)	Conditional variance (or covariance matrix) of *a* given observation *b*
E[a|b]	Conditional expectation of *a* given observations *b*
${\mathrm{round}}_{L}\left(\cdot \right),{\mathrm{mod}}_{L}\left(\cdot \right)$	Lattice round, modulo to a lattice *L* (when it is clear what base region is associated with *L*) (see Section 3.1).
mod(·)	Lattice modulo to the current context’s coarse lattice $L_{c}$’s base region $B_{c}$.

## Appendix A. Proof of Lemma 1, Construction of Mod-Elementary Decoders

**Proof.** Assume $M(A_{j})$ occurs. Then, $M\left(A_{1}\right)⊃\cdots ⊃M\left(A_{j-1}\right)$ have occurred as well. Compute:

(A1)f1U→=moda1†(mod(αkYkn)−W˜d,k)k(A2)=moda1†(mod(αkYkn)−mod(W˜d,k))k(A3)=moda1†(αY→n−W˜d)(A4)=a1†(αY→n−W˜d).

where Equation (A1) is due to the crypto lemma [9] (Theorem 4.1.1), Equation (A2) is since $W_{d,k}^{\prime }\in B_{c}$, Equation (A3) is by Property 4 and Equation (A4) is since $M(A_{1})$ occurred. Now, for any m>1, say $f_{\left[m-1\right]}$ has:

(A5) $$f_{\left[m-1\right]}\left(\vec{U}\right)=A_{\left[m-1\right]}^{†}\left({\vec{\alpha Y}}^{n}-{\tilde{W}}_{d,k}\right).$$

Then, compute (similarly to the base case):

(A6)fmU→=modmodam†(αY→n−W˜d)−…mod[Sm−1am]†A[m−1]†(αY→n−W˜d)+…[Sm−1am]†A[m−1]†(αY→n−W˜d)(A7)=modam†(αY→n−W˜d)−…[Sm−1am]†A[m−1]†(αY→n−W˜d)+…[Sm−1am]†A[m−1]†(αY→n−W˜d)(A8)=modRm−1am†(αY→n+W˜d)+…[Sm−1am]†A[m−1]†(αY→n−W˜d).

Since $M(A_{m})$ has occurred, then ${\left[R_{m-1}a_{m}\right]}^{†}\left({\vec{\alpha Y}}^{n}+{\tilde{W}}_{d}\right)\in B_{c}$, hence by Property 1, Equation (A8) reduces to:

(A9) $$\begin{array}{cc} f_{m}\left(\vec{U}\right) & ={\left[R_{m-1}a_{m}\right]}^{†}\left({\vec{\alpha Y}}^{n}+{\tilde{W}}_{d}\right)+\ldots \\ \; & \;{\left[S_{m-1}a_{m}\right]}^{†}A_{\left[m-1\right]}^{†}\left({\vec{\alpha Y}}^{n}-{\tilde{W}}_{d}\right) \end{array}$$

(A10) $$\begin{array}{cc} \; & =A_{m}^{†}\left({\vec{\alpha Y}}^{n}-{\tilde{W}}_{d}\right). \end{array}$$

Thus, by induction, the described mod-elementary functions behave as desired. □

## Appendix B. Proof of Theorem 1, Asymptotic Lattice Scheme Performance

**Proof.** Using the construction in [23] (Theorem 2), take sequences of lattices $L_{c}^{\left(n\right)},{\left(L_{k}^{\left(n\right)}\right)}_{k\in [K]}$ with base regions $B_{c}^{\left(n\right)},{\left(B_{k}^{\left(n\right)}\right)}_{k}$ where:

- $L_{c}^{\left(n\right)}$ with $B_{c}^{\left(n\right)}$ is good for channel coding in the presence of semi norm-ergodic noise, [23] (Definition 4) scaled to handle semi norm-ergodic [23] (Definition 2) noise of power less than 1.
- Each $L_{k}^{\left(n\right)}\subset L_{c}^{\left(n\right)}$ is *good for mean squared error quantization* [23] (Definition 5), with $\lim_{n}\frac{1}{n}E[∥u_{k}^{\left(n\right)}{∥}^{2}]<2^{-2r_{k}+\varepsilon },$ taking each $u_{k}^{\left(n\right)}∼\mathrm{unif}B_{k}^{\left(n\right)}$ independent.
- Eventually, in *n*, $\frac{1}{n}\log(|L_{c}^{\left(n\right)}\cap L_{k}^{\left(n\right)}\left|\right)<r_{k}.$

For each k∈[K] take $Z_{k}^{n}:=\alpha_{k}Y_{k}^{n}-{\tilde{W}}_{d,k}$, where ${\left({\tilde{W}}_{d,k}\right)}_{k\in [K]}$ is distributed as ${\left(u_{k}^{\left(n\right)}\right)}_{k\in [K]}$ and is independent of ${\left(Y_{k}^{n}\right)}_{k\in [K]}$. Each event $M(A_{j})$ has associated with it some linear combination $R_{j-1}=I$ if j=1 or as in Definition 3 otherwise. Then, by [13] (Appendix V), the linear combination ${\left(R_{j-1}a_{j}\right)}^{†}{\left(Z_{k}^{n}\right)}_{k\in [K]}$ probably lands in the base of a lattice cell as long as that lattice is good for coding for powers greater than $p=\frac{1}{n}E[∥{\left(R_{j-1}a_{j}\right)}^{†}{\left(Z_{k}^{n}\right)}_{k\in [K]}{∥}^{2}]={\left(R_{j-1}a_{j}\right)}^{†}C_{\infty }\left(R_{j-1}a_{j}\right)$. Since $L_{c}^{\left(n\right)}$ is *good for channel coding in the presence of semi norm-ergodic noise with power less than* 1 [23] then (by definition) $Z_{k}^{n}$ occurs in $M(A_{j})$ with arbitrarily high probability eventually in *n* if p<1, and with arbitrarily low probability if p>1. The case where p=1 never occurs when using ε>0 that affects $C_{\infty }$ so that every ${\left(R_{j-1}a_{j}\right)}^{†}C_{\infty }\left(R_{j-1}a_{j}\right)$ is irrational (only countable $R_{j-1},a_{j}$ are possible, thus ε small enough always exist). We now demonstrate that, in this situation, among the high-probability events from Definition 3, there are some whose conditions cannot be strengthened. Any event $M(A_{j})$ with probability eventually high and associated projection $R_{j-1}$ either has some vector $a\in Z^{K}$ with a not in $R_{j-1}$’s null space and $\frac{1}{n}E[∥{\left(R_{j-1}a\right)}^{†}{\left(Z_{k}^{n}\right)}_{k\in [K]}{∥}^{2}]<1$, or no such vector a exists. If there is such an a, then, taking $a_{j+1}=a$, the event $M(\left[A_{j},\;a_{j+1}\right])$ will also have eventually high probability. Repeating the argument, such **a**s can only be found up to m<K times: by then, the matrix $A_{j}$ has column basis for all $R^{K}$, or all choice of $a\in Z^{K}$ yields $\frac{1}{n}E[∥{\left(R_{j-1}a\right)}^{†}{\left(Z_{k}^{n}\right)}_{k\in [K]}{∥}^{2}]>1$. The result of this maximal strengthening of $M(A_{j})$’s conditions to $M(A_{j+m})$ has associated with it a projection $R_{-}$ with the property that any integer vector $a\in Z^{K}$ has either a in $R_{-}$’s null space or $\frac{1}{n}E[∥{\left(R_{-}a\right)}^{†}{\left(Z_{k}^{n}\right)}_{k\in [K]}{∥}^{2}]={\left(R_{-}a\right)}^{†}C_{\infty }\left(R_{-}a\right)>1$. Any projections created in such a way from two different events must be equal, since if the first strengthened event’s projection’s null space had a vector the second’s did not, then the second strengthened event could be further strengthened using the vectors in the first event’s sequence. By above, if $R_{-}$ has nonzero range, and if a nonzero subspace *U* does not contain $\left(\mathrm{image}R_{-}\right)$, then some nonzero integer vector $a\in Z^{K}$ away from *U* has $\min_{s\in U}{\left(a-s\right)}^{†}C_{\infty }\left(a-s\right)<1$. Thus, $S=\mathrm{image}(R_{-})$ is the smallest of all subspaces with the property that any $a\in Z^{K}$ has either $\min_{s\in S}{\left(a-s\right)}^{†}C_{\infty }\left(a-s\right)\geq 1$ or a∈S otherwise. Finally apply Lemma 1 and Corollary 2 to any high-probability event associated with projection $R_{-}$. Since the event eventually has arbitrarily high probability, choosing Δ>1 eventually gives expected distortion arbitrarily close to $d_{L}^{2}$. □

## Appendix C. Closed Form for S*_k_*(v)

For exposition, the definition of $S_{k}\left(v\right)$ is shown semantically, as a minimizer, as opposed to in closed form. Its closed form is readily derived:

(A11) =argminu∈Rk(v−A(k)u)†C(v−A(k)u)

(A12) =argminu∈Rk−2Rev†CA(k)u+u†A(k)†CA(k)u

(A13) $$\begin{array}{cc} \; & =\left[\mathrm{pinv}\left(A^{(k)†}CA^{\left(k\right)}\right)A^{(k)†}C\right]v. \end{array}$$

## Appendix D. KP Parameterization

Here, the parameterization of the KP scheme [13], provided in terms of the asymptotic scheme and Lemma 1, is shown. First, fix some $c\in R^{K}$ and an ordered partition $\left(\Theta_{1},\ldots ,\Theta_{p}\right)$ of [K]. With these variable choices, the KP scheme specifies use of A with columns $a_{m}=1_{\Theta_{m}}$. A matrix with this structure is denoted with subscript as $A_{KP}$.

It also specifies receiver scaling coefficients $\vec{\alpha }$ defined in parts over the partition sets. Starting at m=1 and up through m=p, then the components ${\left(\alpha_{k}\right)}_{k\in \Theta_{m}}$ are specified by a minimization problem dependent on the result of previous steps. Take ${\vec{W}}_{Q\infty }∼N\left(0,\Sigma_{Q\infty }\right)$. In addition, take $c_{\Theta_{k}}$ to be the $|\Theta_{k}|$-vector with coefficients taken from c at indices in $\Theta_{k}$. Now, starting at m=1 and up through m=p, define, if possible:

(A14) $$\begin{array}{cc} {\left(\alpha_{k}\right)}_{\Theta_{m}} & :=\sqrt{\frac{1-\mathrm{var}\left(a_{k}^{†}{\vec{W}}_{Q\infty }\right)}{\mathrm{var}\left(c_{\Theta_{m}}\vec{Y}|A_{KP,m-1}\left(\vec{\alpha Y}+{\vec{W}}_{Q\infty }\right)\right)}}c_{\Theta_{m}}. \end{array}$$

If the square-root in Equation (A14) does not exist (i.e., if the numerator is not positive), then a KP scheme with the partition $\left(\Theta_{1},\ldots ,\Theta_{p}\right)$ cannot be designed for specified c and encoder rates $\left(R_{1},\ldots ,R_{K}\right)$. For any c and nonzero rates, there is always some partition that works. For example, a singleton partition works. A well-formed KP scheme guarantees achievable average distortion:

(A15) $${\hat{d}}_{KP}^{2}=\mathrm{var}\left(X|A_{KP}^{†}\left(\vec{\alpha Y}+{\vec{W}}_{Q\infty }\right)\right).$$
